# Comprehensive Analysis of Macrocirculation and Microcirculation in Microgravity During Parabolic Flights

**DOI:** 10.3389/fphys.2020.00960

**Published:** 2020-08-13

**Authors:** Nana-Yaw Bimpong-Buta, Johanna M. Muessig, Thorben Knost, Maryna Masyuk, Stephan Binneboessel, Amir M. Nia, Malte Kelm, Christian Jung

**Affiliations:** ^1^Medical Faculty, Division of Cardiology, Pulmonology, and Vascular Medicine, University Hospital Düsseldorf, Heinrich-Heine-University, Düsseldorf, Germany; ^2^CARID, Cardiovascular Research Institute Düsseldorf, Düsseldorf, Germany

**Keywords:** weightlessness, microcirculation, parabolic flight, microgravity, hemodynamic changes

## Abstract

**Background:**

Profound knowledge about cardiovascular physiology in the setting of microgravity can help in the course of preparations for human space missions. So far, influences of microgravity on the cardiovascular system have been demonstrated, particularly pertaining to venous fluid shifts. Yet, little is known about the mechanisms of these adaptations on continuous macrocirculatory level and regarding the microcirculation.

**Methods:**

Twelve healthy volunteers were subjected to alternating microgravity and hypergravity in the course of parabolic flight maneuvers. Under these conditions, as well as in normal gravity, the sublingual microcirculation was assessed by intravital sidestream dark field microscopy. Furthermore, hemodynamic parameters such as heart rate, blood pressure, and cardiac output were recorded by beat-to-beat analysis. In these settings, data acquisition was performed in seated and in supine postures.

**Results:**

Systolic [median 116 mmHg (102; 129) interquartile range (IQR) vs. 125 mmHg (109; 136) IQR, *p* = 0.01] as well as diastolic [median 72 mmHg (61; 79) IQR vs. 80 mmHg (69; 89) IQR, *p* = 0.003] blood pressure was reduced, and cardiac output [median 6.9 l/min (6.5; 8.8) IQR vs. 6.8 l/min (6.2; 8.5) IQR, *p* = 0.0002] increased in weightlessness compared to normal gravitation phases in the seated but not in the supine posture. However, microcirculation represented by perfused proportion of vessels and by total vessel density was unaffected in acute weightlessness.

**Conclusion:**

Profound changes of the macrocirculation were found in seated postures, but not in supine postures. However, microcirculation remained stable in all postures.

## Introduction

Over the past decades, spaceflight has been a thriving field of scientific interest (Buckey, 1999; Mairesse et al., 2019). In the meantime, hundreds of astronauts have spent months in space, challenged by the circumstances of microgravity. However, striving to space is not without perils looking at in-flight and postflight health risks (Buckey et al., 1996; Lawley et al., 2017). As commercial flights to space will become accessible in the near future, even more humans with different health states will be exposed to microgravity. Even though putative health risks do not seem permanent, understanding more about physiological processes of the human body under these conditions is of great interest (Hubbard and Hargens, 1989; Demontis et al., 2017; Garrett-Bakelman et al., 2019) as good human health is a prerequisite for the success of any space mission (Hubbard and Hargens, 1989; Demontis et al., 2017).

It is evident that there are only a few platforms that harbor the possibility of fruitful experiments in space or else can mimic conditions of space here on earth. In this regard, spaceflight analogs, such as bed rest, head-down tilt at a moderate angle, or water immersion, have been implemented, particularly in scientific settings (Hargens and Vico, 2016). Even so, since a few decades, parabolic flights have equally proven a promising spaceflight analog (Caiani et al., 2009; Petersen et al., 2011; Norsk, 2014; Shelhamer, 2016; Klein et al., 2019).

It is well known that gravitational changes have significant effects on the cardiovascular system, constantly challenging the cardiovascular system in diminishing venous blood return to the heart and therewith altering cardiac outputs, particularly in seated, and upright postures (Norsk et al., 2006). Here, cardiopulmonary and arterial baroreflexes compensate for gravity-induced dropping of blood pressure by induction of vasoconstriction (Norsk et al., 2006). Prior studies have revealed that in microgravity, the effects on the cardiovascular system are somewhat even more prominent (Aratow et al., 1991; Baisch et al., 2000). In microgravity in particular, headward venous fluid shifts have been reported as hydrostatic gradients are abolished and tissue pressures change (Buckey et al., 1993). In this regard, prominent clinical features of microgravity are puffy faces, nasal congestion, headaches, and bird legs due to dehydration of the lower legs (Hargens et al., 1983; Hargens and Richardson, 2009). Buckey et al. (1993) stressed that, in particular, central venous pressure is altered. It has also been described that in the course of acute loss of gravity in weightlessness, substantial fluid shifts are induced, leading to central volume expansion (Fritsch-Yelle et al., 1996; Norsk et al., 2006, 2015). Hargens et al. (1983) documented the acute effects of these fluid shifts with transition into microgravity (Breit et al., 1993; Hargens and Richardson, 2009) as well as in simulated models of weightlessness. Looking at these facts, one has to bear in mind that understanding the mechanism of fluid shifts under these conditions remains complex, particularly as overall fluid response mechanisms may have significant medical implications (Nicogossian et al., 1991; Simanonok and Charles, 1994). As stated above, one of the primary fluid shift mechanisms upon entrance into microgravity is the relocation of vascular fluids to cephalad compartments (White and Blomqvist, 1998; Drummer et al., 2000; Hawkey, 2003). However, it should be mentioned that overall fluid distribution also entails fluid allocations into other locations such as extravascular and extracellular compartments (Leach et al., 1996; Drummer et al., 2000), for instance, in the course of transcapillary fluid shifts (Hargens and Richardson, 2009). Furthermore, shifts of interstitial fluids have been reported in former studies as potentially underlying causes of complex systemic adaptations of the human body to weightlessness (Kirsch and Von Ameln, 1981; Blomqvist, 1983). Another noteworthy aspect of fluid distributions under these conditions is its timescale: previous studies have revealed the importance of characterizing short-term, mid-term, and long-term fluid shift alterations in this setting (Norsk et al., 2015; Gerber et al., 2018; Norsk, 2020), even with respect to postflight readaptations (Moore and Thornton, 1987).

Furthermore, details about general physiologic adaptations during spaceflights have been addressed (Tipton and Hargens, 1996). These changes occur immediately upon entering microgravity and last for at least several days or even weeks (Norsk et al., 2006, 2015). As an excellent health status is a prerequisite of any astronaut, candidates for space missions undergo profound prior medical testing proving excellent health states. Thus, most astronauts recover from postflight health deficits in a timely fashion of a few weeks, and permanent health deficiencies have remained scarce. However, postflight cardiovascular deconditioning, as in orthostatic intolerance, is an important issue. Buckey et al. (1996) and Lee et al. (2015) have investigated about its clinical relevance and implications (Buckey et al., 1996; Lee et al., 2015).

So far, scientific works have been focusing on macrocirculatory parameters such as blood pressure, cardiac output, and heart rate during human space missions as well as with spaceflight analogs (Mukai et al., 1991; Fritsch-Yelle et al., 1996; Schlegel et al., 1998; Norsk et al., 2006, 2015; Caiani et al., 2009; Coupé et al., 2009). However, in-flight measurements of these cardiovascular parameters are difficult to obtain, and results have been inconsistent (Fritsch-Yelle et al., 1996; Norsk et al., 2006; Verheyden et al., 2009; Petersen et al., 2011; Klein et al., 2019).

With respect to the known data about cardiovascular adaptations in space, alterations of the microcirculation under these conditions seem likely, but the impact of acute weightlessness on the microcirculation has not yet been addressed. The microcirculation is regarded the largest part of the circulation. It entails a large network of arterioles and venules that facilitates gas and nutrient exchange on tissue and endothelial levels (Coupé et al., 2009), with an estimated surface area of 350 m^2^. In this regard, the microcirculation plays a crucial role in blood flow regulations, ensuring adequate organ function (Jung et al., 2016a). The evaluation of the microcirculation has become more and more clinically relevant, in particular, in the setting of critically ill patients (De Backer et al., 2014; Jung et al., 2016b). In these scenarios, the microcirculation has been identified as one of the key predictors of mortality. The timely evaluation of the microcirculation has been esteemed one of the tools for improvement of therapeutic strategies. Former studies demonstrated that in challenging scenarios with profound alterations of the cardiovascular system, macrocirculation, and microcirculation might differ substantially in responses (Jung et al., 2010; Jung et al., 2016b). Therefore, in our setting, one of the aims was to investigate about differences of macrocirculation and microcirculation as substantial effects on the cardiovascular system are generally known. Handheld video microscopes, as the one we implemented in our setting, have been classified helpful in the assessment of microcirculatory flow (Massey and Shapiro, 2016; Ince et al., 2018).

There were two aims of this study: (1) to test the feasibility of measurements of the sublingual microcirculation during parabolic flight maneuvers as a novel approach on a spaceflight-mimicking platform and (2) to assess alterations of hemodynamic cardiovascular parameters of the macrocirculation and the microcirculation during microgravity and to evaluate the effect of supine and seated body postures on these variables.

## Materials and Methods

### Study Population

Twelve healthy volunteers (seven male, median age of the whole group 29 years) were recruited for this study. Airworthiness (proven by medical certificate) was attested prior to participation in this study. The study was conducted in accordance with the Declaration of Helsinki (1975, revised in 2008), and the protocol was approved by the German Ethics Committee of the Medical Faculty of the University Hospital Duesseldorf, Germany (Date of approval: August 14, 2017; Project Identification number: 2017054297), and by the French Ethics Committee [Comité de Protection des Personnes (CPP) Nord-Ouest III] of the Medical Faculty of the University of Caen (Date of approval: September 6, 2017; Project Identification number: 2017-A01185-48). Written informed consent was voluntarily provided by all participants of the study.

### Parabolic Flight

The study was conducted within a participation in a so-called parabolic flight campaign by the German space agency [Deutsches Zentrum für Luft-und Raumfahrt (DLR)] as described previously (Bimpong-Buta et al., 2018a). The location of this campaign was in Bordeaux (France) with flight over the Mediterranean Sea and the Atlantic Ocean. On-site in Bordeaux, the French company NoveSpace (headquarter in Mérignac, France) was in charge of regulations of adequate aviation procedures. On each flight day, 31 parabolic flight maneuvers were performed. The aircraft implemented in this flight campaign was an Airbus 310. To obtain best parabolic flight trajectories, the aircraft was aviated by well-trained jet pilots. In the course of each parabolic flight path, alternating states of gravity can be experienced aboard. These gravity states range from earthly gravity (1 G) to begin with (“steady flight”) followed by a state of hypergravity (“1.8 G pull-up”) followed by a state of microgravity (0 G) for the duration of 22 s. Hereafter, *via* a second phase of hypergravity (“1.8 G pull-out”), regular gravity (1G) is resumed at the end of each parabolic flight maneuver. Details about the flight maneuver have been published before (Schlegel et al., 1998; Shelhamer, 2016).

One of the primary concerns in the course of preparation of the flight campaign was the possible occurrence of motion sickness due to the anticipated abrupt gravitational changes inherent to the scheduled flight maneuvers. In worst-case scenarios, health issues of the crew or participants might have led to cancelation of a flight day. With respect to the extensive efforts of preparation of each experiment prior to the campaign, one aim prior to takeoff was to minimize possible interferences or interruptions of the flight maneuvers, especially as this physiologic reaction has proven foreseeable. On the basis of experiences from former flight campaigns, the intentional application of antiemetic medications prior to takeoff has helped alleviate this issue. Thus, in order to prevent motion sickness during the parabolic flight maneuvers, the antiemetic drug scopolamine was administered subcutaneously around 2 h prior to takeoff on a voluntary basis. In one of our previous studies, we could demonstrate that scopolamine does not affect our measurements of the sublingual microcirculation (Bimpong-Buta et al., 2018b). Nonetheless, one has to bear in mind that the application of scopolamine in this setting might have unknowingly modified the results of the performed measurements in other ways (see also limitations).

### Experimental Setup

In our experimental setup, for each flight day, three test subjects were scheduled. As 31 parabolas were flown per flight day, each test subject was examined in the course of 10 consecutive parabolas, with five parabolas in the supine posture and five parabolas in the seated posture. Each data set comprised the measurements of parameters of microcirculation and parameters of macrocirculation, respectively.

### Macrocirculation

The macrocirculation was investigated using a device for continuous and non-invasive beat-to-beat measurement of hemodynamic blood flow (CNAP^®^ Monitor 500 HD, CNSystems Medizintechnik GmbH, Graz, Austria). In short, the analysis includes blood pressure wave form documentation and provides derived parameters. The device has been validated in clinical trials indicating an excellent comparability with invasive measurements (Smolle et al., 2015; Wagner et al., 2015; Rogge et al., 2017). In the course of preparations of the project, a specially trained CNAP^®^ instructor was invited from Austria for hands-on training sessions with our team of operators. With respect to the planned rotations of the test subjects aboard and therewith indicated changes of postures, this training focused on optimization of procedures of (re-) calibrations of the device to ensure correct measurements throughout the experiment. For correct measurements, the calibration entailed positioning the left hand on the chest. Aboard, all beat-to-beat data were stored with defined markers for later analysis. For quick setup, the monitor was connected to each test subject *via* a single-line finger sensor placed on the right index finger. Before each set of measurements, notably after the change of postures, the monitor was calibrated and checked to ensure proper function in all its particulars. In this manner in our setup, for each parabola, the following parameters were obtained: blood pressure (BP), heart rate (HR), stroke volume (SV), cardiac output [(CO) = HR × SV], cardiac index [(CI) = CO/body surface area (BSA)], and systemic vascular resistance {(SVR) = [80 × (mean arterial pressure – mean right pressure)]/CO}.

### Microcirculation

The microcirculation was assessed by implementation of the sidestream dark field microscopy (MicroScan^®^ device, Microvision Medical, Amsterdam, Netherlands) as described before (Ince, 2005; De Backer et al., 2007; Bimpong-Buta et al., 2018b). This intravital microscope is designed for real-time measurement of the human sublingual microcirculation. In more than 200 clinical studies, it has been proven to serve as a valid diagnostic tool for high-quality imaging of the sublingual capillary network. With a highly sensitive camera at the tip of the device, real-time recordings of the sublingual capillary network can be performed and visualized on a tablet screen to be saved for later analysis. In this regard, in our setting, the device was mounted on the side of the tongue of each participant with application of gentle pressure to ensure just sufficient contact of the tip of the device with the sublingual surface. As part of the visualization software, the monitor offered a real-time feedback about the quality of the intended recording. Thus, high-quality visualization of the microcirculatory network could be ensured.

The tablet we utilized in this setting is the Microsoft Surface Pro 4 (Redmond, Washington, United States). After acquisition of the imagery data, a device-specific software (AVA, Version 4.3 C) is implemented for data analysis. For the evaluation of the microcirculation, for each parabola, the following parameters were measured: Proportion of perfused vessels [(PPV) = 100 × (total number of perfused vessels/total number of vessels)], perfused vessel density [(PVD) = total length of perfused vessels divided by the analyzed area], total vessel density [(TVD) = total number of vessel crossings], number of crossings [(NC) = number of vessel intersections the lines in a grid of 3 equidistant horizontal and vertical lines], and the perfused number of crossings [(PNC) = number of vessel crossings with continuous flow].

### Statistical Analysis

Statistical analysis was performed applying a commercially available software (GraphPad Prism Software, Version 6, GraphPad Software, San Diego, CA, United States). As the size of the group of test subjects was rather small, we did not assume a Gaussian distribution in this setting. The data are presented as median in the course of repeated measures. In this regard, the statistical tests applied were the Mann–Whitney test and the Friedman test, respectively. In the course of *post hoc* analysis, the Dunn’s multiple comparisons test was implemented. A two-tailed *p*-value < 0.05 was considered statistically significant.

## Results

**FIGURE 1:**
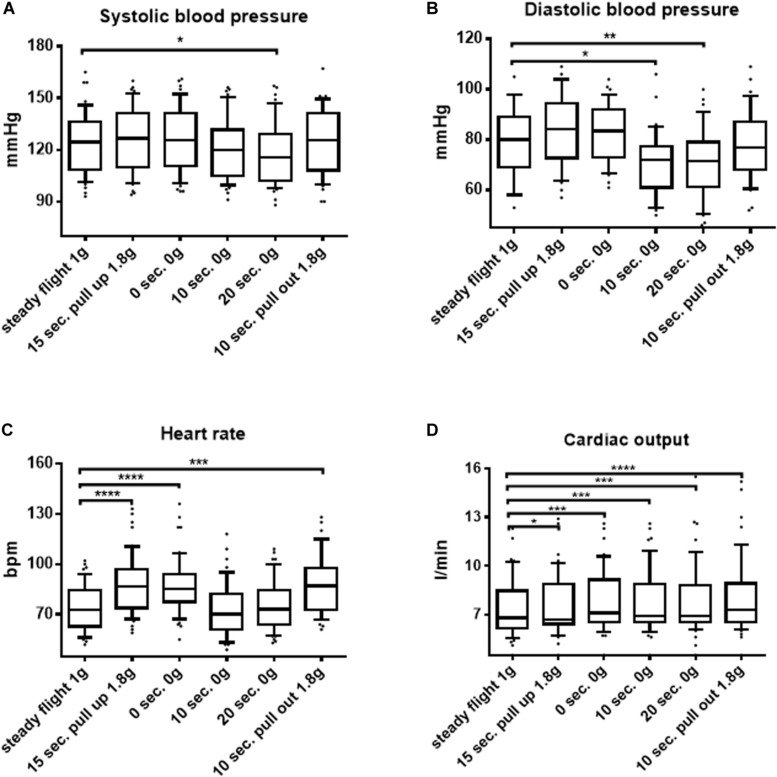
Macrocirculatory parameters in the course of parabolic flights in the seated position. **(A)** Systolic blood pressure, **(B)** diastolic blood pressure, **(C)** heart rate, and **(D)** cardiac output changes during the course of a parabola in the seated position are shown. Data are presented as median and interquartile range (IQR). Significant differences between groups are shown (Friedman test and Dunn’s multiple comparisons *post hoc* test). ^∗^*p* < 0.05, ^∗∗^*p* < 0.01, ^∗∗∗^*p* < 0.001, and ^∗∗∗∗^*p* < 0.0001.

The baseline characteristics of the study population are shown in Table 1. Throughout the course of each parabola in seated postures, significant changes of hemodynamic parameters reflecting the macrocirculation could be observed. Thus, systolic as well as diastolic blood pressure decreased in weightlessness. Blood pressures reached a median of 116 mmHg (102; 129) interquartile range (IQR) vs. 125 mmHg (109; 136) IQR, *p* = 0.01, e.g., a median of 72 mmHg (61; 79) IQR vs. 80 mmHg (69; 89) IQR, *p* = 0.003, respectively, after 20 s of microgravity in the seated posture (Figure 1). Furthermore, in the seated posture, cardiac output increased in 0 G as well as in hypergravity compared to steady flight values with a median of 6.9 l/min (6.5; 8.8) IQR vs. 6.8 l/min (6.2; 8.5) IQR, *p* = 0.0002, upon 20 s of weightlessness and of 7.3 l/min (6.5; 8.9) IQR, *p* < 0.0001 after 10 s of 1.8 G in the pull-out phase of the parabola, as shown in Figure 1. Interestingly, heart rate increased during phases of hypergravity up to median values of 87 bpm (74; 97) IQR after 15 s of hypergravity in the pull-up phase compared to steady flight values with a median of 73 bpm (63; 85) IQR, *p* < 0.0001, whereas heart rate returned to baseline levels with median values of 73 bpm (64; 84) IQR after 20 s of microgravity (Figure 1). Stroke volume decreased in microgravity, whereas systemic vascular resistance was unchanged in weightlessness in seated as well as in supine postures (data not shown).

**TABLE 1:** Baseline characteristics of the study population. Data are presented in counts or as median and IQR.

*n*	12
Sex	7 male, 5 female
Age (years)	29 [23–31]
Height (m)	1.77 [1.71–1.90]
Weight (kg)	80 [62–90]
BMI (kg/m^2^)	24.5 [20–25]
BSA (m^2^)	2 [1.72–2.13]
BP sys (mmHg)	110 [106–127]
BP mean (mmHg)	92 [87–94]
BP dia (mmHg)	78 [69–81]
HR (bpm)	79 [61–95]

*BMI, body mass index; BSA, body surface area; BP, blood pressure, HR, heart rate; and IQR, interquartile range.*

**FIGURE 2:**
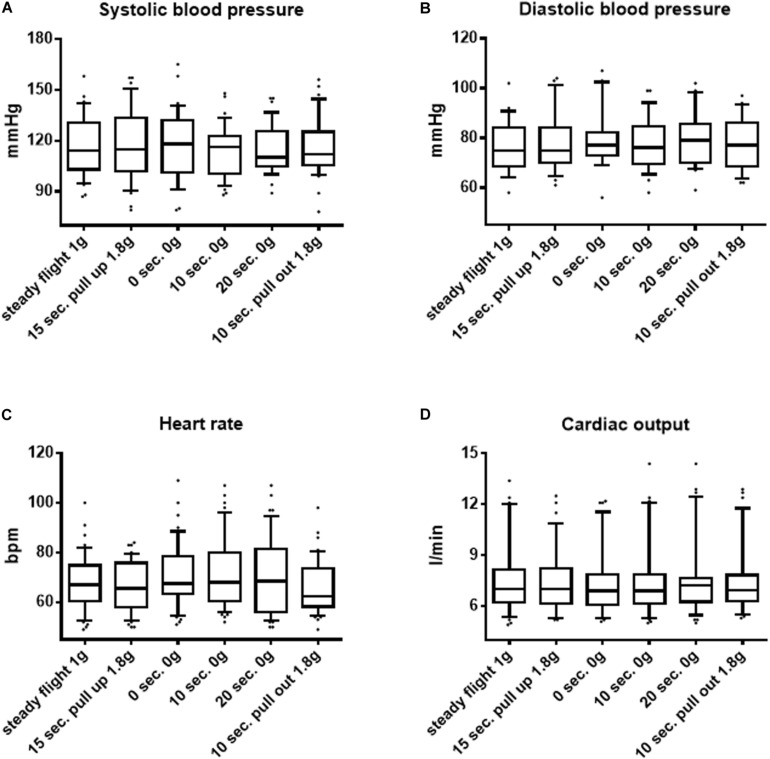
Macrocirculatory parameters in the course of parabolic flights in the supine position. **(A)** Systolic blood pressure, **(B)** diastolic blood pressure, **(C)** heart rate, and **(D)** cardiac output changes during the course of a parabola in the supine position are shown. Data are presented as median and interquartile range (IQR). Significant differences between groups are shown (Friedman test and Dunn’s multiple comparisons *post hoc* test). ^∗^*p* < 0.05, ^∗∗^*p* < 0.01, ^∗∗∗^*p* < 0.001, and ^∗∗∗∗^*p* < 0.0001.

In contrast to the macrocirculatory changes observed in seated postures throughout the course of each parabola, in supine postures, no significant alterations regarding blood pressure, heart rate, or cardiac output could be observed, as shown in Figure 2.

**FIGURE 3:**
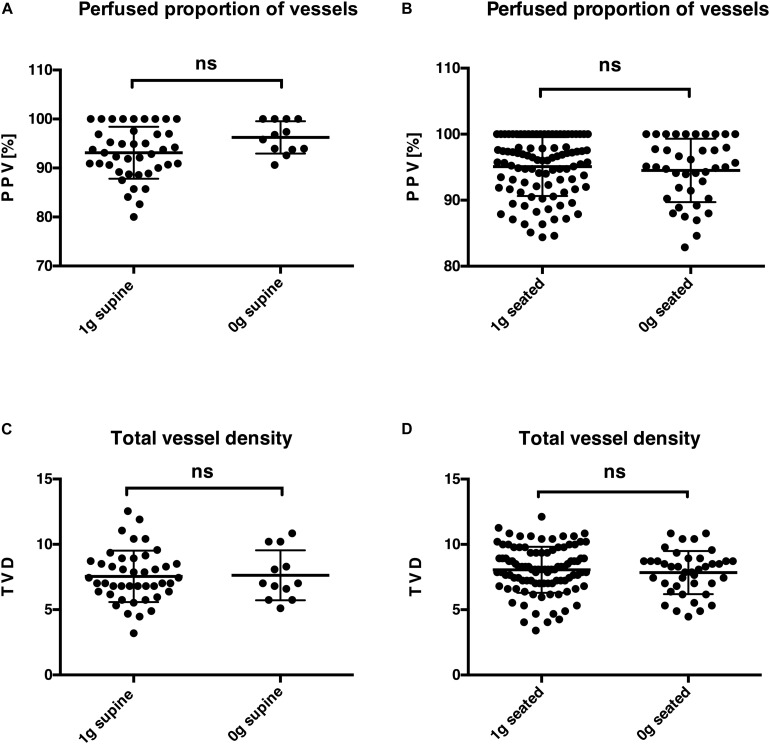
Microcirculation in steady flight and acute weightlessness. Comparison of obtained parameters for normal gravity (1 *G*) and microgravity (0 *G*) for two variables of sublingual microcirculation, namely, the perfused proportion of vessels [**(A)** in the supine posture and **(B)** in the seated posture] and the total vessel density [**(C)** in the supine posture and **(D)** in the seated posture]. The test applied in statistical analysis was the Mann–Whitney test. ns, not significant.

Visualization and recording of high-quality imaging of the sublingual capillary network with a highly sensitive intravital sidestream dark field microscope connected to a tablet screen were feasible in microgravity and during steady flight phases. Despite the observed impact of parabolic flight maneuvers on the macrocirculation, on the level of microcirculation, no significant alterations could be detected in parabolic flight maneuvers in supine and seated postures, as shown in Figure 3A: PPV (%): 1 *G* supine [median of 93 (90; 97) IQR] vs. 0 *G* supine [96 (94; 100) IQR], *p* = 0.07; Figure 3B: PPV (%): 1 *G* seated [96 (92; 100) IQR] vs. 0 *G* seated [95 (90.5; 99.5) IQR], *p* = 0.57; Figure 3C: TVD: 1 *G* supine [7 (6; 9) IQR] vs. 0 *G* supine [7 (6; 10) IQR], *p* = 0.92; and Figure 3D: TVD: 1 *G* seated [8.1 (7; 9.35) IQR] vs. 0 *G* seated [8.2 (6.9; 8.7) IQR], *p* = 0.63.

## Discussion

The aims of the presented study were to investigate the impact of acute weightlessness as well as of acute hypergravity on macro-hemodynamic parameters and on the sublingual microcirculation in the setting of parabolic flight maneuvers as an established spaceflight analog (Shelhamer, 2016). Moreover, we were interested in learning more about responses of the cardiovascular system in seated postures in comparison to supine postures under these conditions. So far, gravitational loads have had the most prominent effects on the cardiovascular system in upright postures. This is evident as in upright postures that the weight of the blood column is more prominent than in supine postures. Thus, upon microgravity exposure with abrupt loss of the effect of the blood column, more intense alterations are expected in upright postures due to abrupt loss of the weight of the blood column. On the other hand, for supine postures, with primarily abolished vertical weight of the blood column, even upon entrance into microgravity, effects would be anticipated to remain low. Bearing these thoughts in mind, we did not expect high alterations of cardiovascular parameters in supine postures.

As a novel approach, we implemented continuous beat-to-beat-analysis and visualization of the microcirculation with a handheld video microscope to monitor the sublingual microcirculation under these conditions. Prior works in the setting of septic and cardiogenic shock underlined the fact that, particularly in these life-threatening states with high mortality rates, assessment of the cardiovascular status can be challenging as tracked macrocirculatory parameters may seem stable enshrouding deleterious microcirculatory disorders causative for organ dysfunctions (Jung et al., 2016a; Jung, 2019; Legrand et al., 2019). The evaluation of microcirculation at the bedside has been esteemed highly important (Kara et al., 2016). In particular in these settings, the assessment of the sublingual microcirculation with handheld videos microscopes has proven feasible and reliable (De Backer and Dubois, 2001; Ince et al., 2018).

In our current experiment, we demonstrated that measurement of sublingual microcirculation using intravital sidestream dark field microscopy is feasible in microgravity. In line with prior studies, our work demonstrates that acute weightlessness significantly affects the cardiovascular system. In our setting, significant alterations of macrocirculatory parameters such as blood pressure and cardiac output were evident in seated but not in supine postures, as anticipated. Interestingly, the microcirculation remained unchanged in the course of the parabolic flight maneuvers regardless of body posture.

For the acute setting, previous studies conducted during human space missions and spaceflight analogs on earth have reported inconsistent data regarding the changes of cardiovascular parameters under these conditions (Fritsch-Yelle et al., 1996; Petersen et al., 2011; Norsk, 2014; Klein et al., 2019). For instance, Klein et al. (2019) documented decreases of mean arterial pressures, whereas Norsk et al. (2006) constituted no changes of mean arterial pressures in the same setting. These discrepancies might be caused by the fact that blood pressure evaluation under baseline conditions in some studies was done in the standing or seated posture, whereas in other studies, blood pressure was measured in the supine posture. This might have affected on account of differing references. In our setting, baseline parameters for all variables were documented under normal gravity (1 G) and for both tested body positions (seated and supine) to ensure correct correlations to baseline references. Another reason for differing results for macrocirculatory parameters among diverse studies pertaining to short-term microgravity during parabolic flight maneuvers might be different techniques for blood pressure measurements. In our setting, hemodynamic variables were obtained by beat-to-beat analysis closely linked to a defined time line. Thus, great time resolutions could be provided. Our findings confirmed those of Verheyden et al. (2009) with decrease of blood pressures during space missions with more prominent effects for upright postures in comparison to the effects measured in supine postures. However, these results contrast the findings of Norsk et al. in other studies that showed that blood pressure was unchanged in parabolic flight maneuvers (Norsk et al., 2006) but reduced during space missions (Norsk et al., 2015), even though Norsk (2020) performed their baseline measurements in supine postures as we did.

Another cardiovascular parameter we looked at is the heart rate. Here, we found an increase in heart rate during phases of hypergravity in seated but not in supine postures, whereas heart rate remained unchanged during phases of microgravity compared to baseline values. However, previous studies reported a decrease in heart rate in weightlessness (Fritsch-Yelle et al., 1996; Hughson, 2009). In our setting, these decreased heart rates might have been overridden on account of the rapid changes between hypergravity and microgravity in the course of repeated parabolic flight maneuvers. On the other hand, the increase of heart rate we documented might have been reactive to diminution of peripheral resistance upon entrance into microgravity, accounting for the increase of cardiac output. Thus, heart rate might have primarily fueled the compensatory mechanism to counteract fluid shifts and blood pressure alterations. These findings are in line with findings of previous studies (Norsk et al., 2006, 2015) as we found an increase in cardiac output in acute weightlessness in seated postures compared to measurements performed in normal gravity. This increase in cardiac output is furthermore caused by increased central blood volume (Norsk et al., 2006, 2015) as previous echocardiographic studies showed a distension of the heart chambers in this process (Videbaek and Norsk, 1996; Pump et al., 1999; Caiani et al., 2009). Again, in supine postures, we could not detect any significant differences in cardiac output when comparing normal gravity, microgravity, and hypergravity.

In acute settings, possible discrepancies of macrocirculatory and microcirculatory measurements are known (Jung et al., 2009). Accordingly, in our study, despite observed significant consequences of altering gravitational loads on the parameters of macrocirculation, the sublingual microcirculation remained unchanged, regardless of body posture, or gravitational state. This investigation of the sublingual microcirculation is a novel approach as, to our knowledge, there are no data thereabout to date. However, previous studies looked at other circulatory networks of the human body. In the assessment of putative health risks for astronauts, visual impairment has been documented (Zhang and Hargens, 2014; Zhang and Hargens, 2018). Lawley et al. (2017) constituted that in prolonged stays in microgravity, slightly elevated intracranial pressures might be causative for remodeling of the eye but intracranial pressures were reduced in acute microgravity. Other studies about cerebral autoregulation showed that cerebral autoregulation and perfusion are not altered in weightlessness or head-down bed rest (Arbeille et al., 2001; Iwasaki et al., 2007). Thus, it is tempting to speculate that the human organism somewhat harbors safety mechanisms to counteract hemodynamically challenging scenarios such as weightlessness aiming at preserving steady organ perfusion to ensure nutrient and gas exchange. In this regard, localized vascular adaptations of the sublingual vascular beds might explain the stability of sublingual flow, even in microgravity. Possibly, pre-capillary sphincters and myogenic responses could contribute to this process. As previous studies have demonstrated, vasoactive hormones such as elevated aldosterone levels (Limper et al., 2014) might additionally contribute to vasoconstriction of arterioles. In contrast to our finding of unaltered microcirculation in the setting of acute weightlessness, a previous study by Coupé et al. (2009) indicated a microvascular dysfunction upon 56 days of head-down bed rest, as another spaceflight analog. In that study, as a marker of microcirculatory function, the endothelial dysfunction was measured, whereas our approach entailed direct visualization of the sublingual microcirculation with subsequent software-based calculation of microvasculatory parameters. Taking these differences into account, these studies might not be adequately comparable.

Certain limitations of our study have to be addressed: Parabolic flights have been known as established spaceflight analog for emulation of acute states of microgravity. However, as with other spaceflight analogs, there might be differences to prolonged states of microgravity during human space missions. In this regard, measurements of cardiovascular parameters over longer periods might differ from our measurements of acute alternating gravitational loads with rather short periods of weightlessness of a little more than 20 s per aviated parabola.

As motion sickness during the parabolic flight maneuvers has been a common side effect in former flight campaigns, application of scopolamine remains a strong recommendation for all flight participants. Frankly, there was no strong scientific justification for its use. On the other hand, in our pre-study experiment prior to the campaign, we could show that use of scopolamine does not interfere with our measurements of sublingual microcirculation (Bimpong-Buta et al., 2018b). However, one has to admit that other known effects of scopolamine, in particular on the cardiovascular system, might have altered our results. On the other hand, scopolamine has an elimination half-life of approximately 2 h (Stetina et al., 2005), which might have mitigated its subsequent effects on our measurements, as scopolamine was administered around 2 h prior to takeoff.

Another point is that one has to bear in mind that the sublingual microcirculation harbors tissue-specific responsiveness that does not necessarily represent all microcirculation of the human body, so that findings in this particular vascular bed cannot necessarily be transferred to all other vascular beds. However, as described earlier, in former studies, the microcirculation could serve as a reliable predictor of mortality outcomes and may gain further importance in future studies as a tool of improvement of therapeutic strategies, particularly for critically ill patients.

Finally, it should be noted that the CNAP monitor was calibrated multiple times in the course of our data acquisition, as mentioned above. Reasonable accuracy of its measurements has been proven (Wagner et al., 2015; Rogge et al., 2017). The intention of repeated calibrations was to ensure correct measurements throughout the experiment. However, possible impacts of macrocirculatory and microcirculatory changes on the accuracy of the instrument cannot be fully excluded.

## Conclusion

In summary of our works, as a novel approach, we could demonstrate that the measurement of the sublingual microcirculation in microgravity induced by parabolic flight maneuvers is feasible. Our results underline the fact that profound alterations of the macrocirculatory hemodynamic parameters occur under these circumstances. In our setting, these alterations were most prominent in seated postures but not evident in supine postures, as could be anticipated with regard to known abolished weight of blood columns in supine postures. However, microcirculation remained stable in acute weightlessness regardless of body posture, suggesting that localized and tissue-specific reactions in this vascular bed might be causative. For instance, it would be interesting to examine standing participants with a similar setting to investigate about possible effects of a more prominent blood column in standing postures. Here, we would expect more prominent results of the documented differences as the weight of the blood column in standing humans is again more than for seated postures.

Future studies on cardiovascular parameters seem warranted to learn more about physiologic circulatory response mechanisms under these conditions. This could be helpful for establishments of health safety strategies, as the good health of humans in space will remain a primary concern for upcoming commercial and scientific endeavors.

## Data Availability Statement

All datasets generated for this study are included in the article/Supplementary Material.

## Ethics Statement

The studies involving human participants were reviewed and approved by Heinrich-Heine-University. The patients/participants provided their written informed consent to participate in this study.

## Author Contributions

All authors listed have made a substantial, direct and intellectual contribution to the work, and approved it for publication.

## Conflict of Interest

The authors declare that the research was conducted in the absence of any commercial or financial relationships that could be construed as a potential conflict of interest.

## Supplementary Material

FIGURE S1 Macrocirculatory parameters in the course of parabolic flights: seated and supine positions in comparison. **(A)** Systolic blood pressure, **(B)** Diastolic blood pressure, **(C)** Heart Rate, and **(D)** Cardiac Output. As an overview here, the Figures 1, 2 have been combined, for easier apprehension of the outcomes of differing postures.

## Abbreviations

0 G: microgravity
1 G: regular terrestrial gravity
1.8 G: hypergravity
BP: sys/dia systolic/diastolic blood pressure
bpm: beats per minute
BSA: body surface area
HR: heart rate
IQR: interquartile range
PPV: perfused proportion of vessels
TVD: total vessel density.
