# Supplementary figures and images for: Comprehensive Analysis of Macrocirculation and Microcirculation in Microgravity During Parabolic Flights
Source: Front Physiol. 2020 Aug 13;11:960. doi: 10.3389/fphys.2020.00960 (PMC7438475; doi:10.3389/fphys.2020.00960)

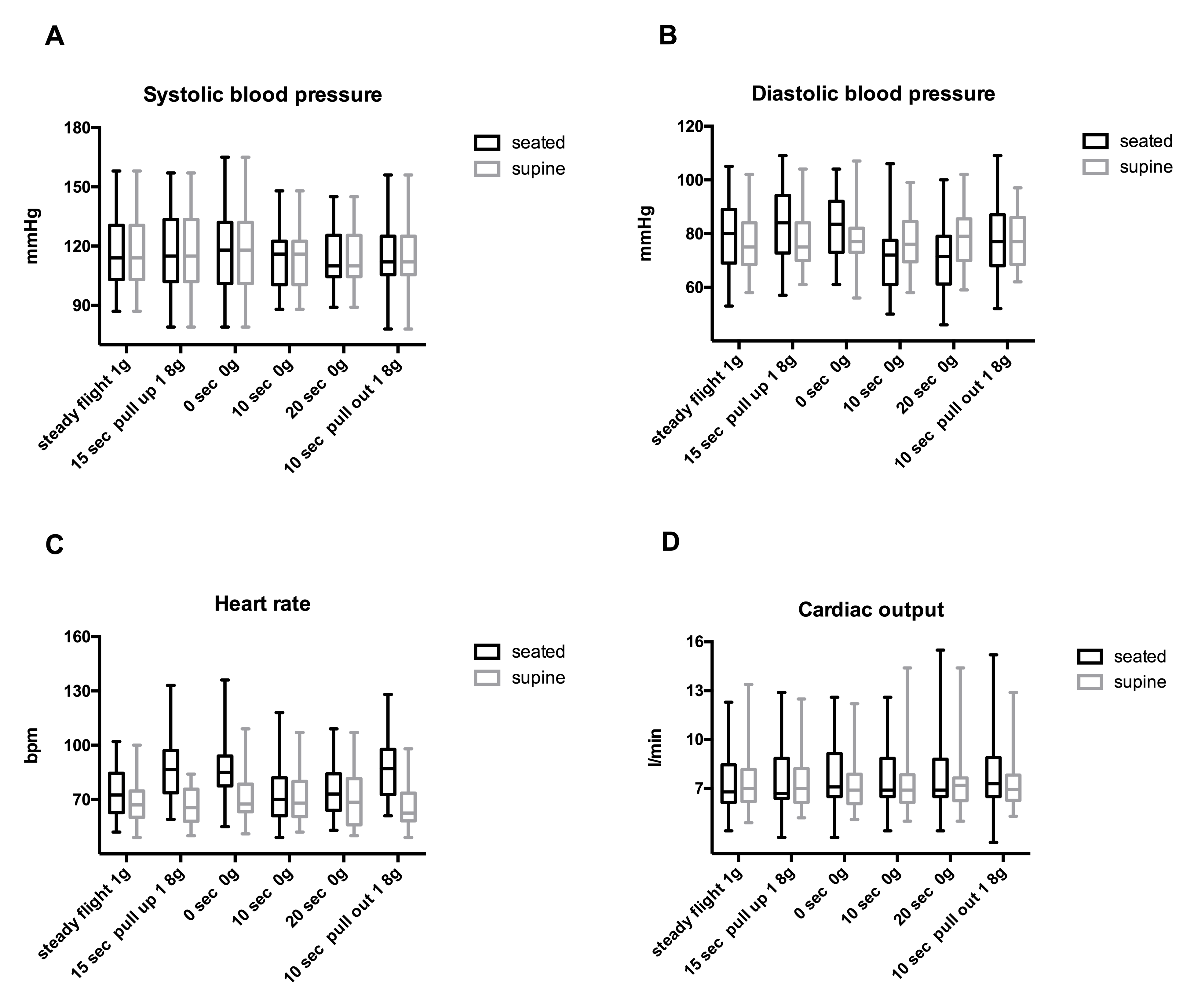

Supplement: FIGURE S1 — Macrocirculatory parameters in the course of parabolic flights: seated and supine positions in comparison. (A) Systolic blood pressure, (B) Diastolic blood pressure, (C) Heart Rate, and (D) Cardiac Output. As an overview here, the Figures 1, 2 have been combined, for easier apprehension of the outcomes of differing postures. [file Image_1.tiff]
